# DOES WEARING AUGMENTED-REALITY GOGGLES AFFECT OLDER ADULTS’ KINEMATICS DURING GAIT?

**DOI:** 10.1093/geroni/igz038.1226

**Published:** 2019-11-08

**Authors:** Edgar R Vieira, Jansen Estrazulas, Fernanda Civitella, Jorge Carreno, Newton D’Souza, Ebru Ozer, Francisco Ortega

**Affiliations:** 1 Department of Physical Therapy, Florida International University, Miami, Florida, United States; 2 College of Health, Amazonas State University, Manaus, Amazonas, Brazil; 5 Department of Interior Architecture, Florida International University, Miami, Florida, United States; 6 Department of Landscape Architecture + Environmental and Urban Design, Florida International University, Miami, Florida, United States; 7 Department of Computer Science, Colorado State University, Fort Collins, Colorado, United States

## Abstract

Virtual-reality (VR) testing can cause motion sickness and impair safety, especially for older adults, but augmented-reality (AR) may allow the testing of holograms embedded into a mixed-reality environment without the VR impediments. However, wearing AR googles may affect the way people walk, but this possibility has not being tested. The objective of this study was to evaluate if wearing AR googles during gait would affect the kinematics of older adults. Ten older adults (68±5 years), who could walk without assistive devices, participated in this study. The participants walked outdoors in a public park with and without the AR googles. The participants were instrumented inertial movement units to track their kinematics (MTw Awinda trackers, Xsens Technologies B.V., Enschede, the Netherlands). The goal of the study was to assess if simply wearing the googles would affect gait, therefore no holograms were displayed. Ten gait cycles were analyzed and the mean of each subject was used to compare the joint kinematics between the conditions (with vs without googles) using T-tests in SPSS 18. The foot, ankle, knee and hip angles were not different between the conditions (p>0.05), but there were significantly less trunk flexion at 44% of the gait cycle (p=0.035) and less forward head flexion throughout the gait cycle (p=0.023) when the participants were wearing the googles vs. when they were not. The findings indicate that wearing AR goggles changed the trunk and head posture cycle, but did not affect the lower limb kinematics during gait.

